# A Novel Cloning Template Designing Method by Using an Artificial Bee Colony Algorithm for Edge Detection of CNN Based Imaging Sensors

**DOI:** 10.3390/s110505337

**Published:** 2011-05-17

**Authors:** Selami Parmaksızoğlu, Mustafa Alçı

**Affiliations:** Electrical and Electronics Engineering, Engineering Faculty, Erciyes University, Turkey; E-Mail: malci@erciyes.edu.tr

**Keywords:** cellular neural networks, edge detection, artificial bee colony algorithm, real time imaging sensors

## Abstract

Cellular Neural Networks (CNNs) have been widely used recently in applications such as edge detection, noise reduction and object detection, which are among the main computer imaging processes. They can also be realized as hardware based imaging sensors. The fact that hardware CNN models produce robust and effective results has attracted the attention of researchers using these structures within image sensors. Realization of desired CNN behavior such as edge detection can be achieved by correctly setting a cloning template without changing the structure of the CNN. To achieve different behaviors effectively, designing a cloning template is one of the most important research topics in this field. In this study, the edge detecting process that is used as a preliminary process for segmentation, identification and coding applications is conducted by using CNN structures. In order to design the cloning template of goal-oriented CNN architecture, an Artificial Bee Colony (ABC) algorithm which is inspired from the foraging behavior of honeybees is used and the performance analysis of ABC for this application is examined with multiple runs. The CNN template generated by the ABC algorithm is tested by using artificial and real test images. The results are subjectively and quantitatively compared with well-known classical edge detection methods, and other CNN based edge detector cloning templates available in the imaging literature. The results show that the proposed method is more successful than other methods.

## 1. Introduction

Chua and Yang introduced the Cellular Neural Network (CNN) model, which enables new possibilities in signal processing and which can be appropriately implemented as an integrated circuit. The CNN is formed in a way that the cells are connected in a two-dimensional (2D) network structure, and it is also able to make simultaneous signal processing [1,2]. In the network structure, a cell is connected with only the neighboring cells by means of a certain set of parameters. The set of these parameters determines the dynamic behavior of the CNN and are called “cloning template”. The two dimensional network architecture of CNNs provides a convenient structure for image processing applications and real time imaging sensors and, therefore, the major application field of CNNs is image processing [3,4]. Because of the faster image processing ability of CNN-based imaging sensors and circuits, complex image processing tasks can also be accomplished successfully. Several complex image processing applications have been successfully carried out through CNNs [5–11]. For realizing complex image processing applications based on the CNN structure, preliminary image processing techniques such as edge detection, diffusion and dilation must be used. Therefore the overall performance of such task depends strongly on the quality of the preliminary process.

Edge detection is a very important area in the field of computer vision, due to the fact it is used as a main task or preliminary task for complex image processing techniques such as segmentation, registration and object recognition and identification. An ideal edge detection process is finalized by an image which is formed as the set of connected curves of the objects boundaries in an image. The main indicators demonstrating the quality of edge detection are continuous line detection of the details and boundaries of the objects, the thinness of the lines and the lack of noise in edge detected image. Edge detection processes have been widely studied in the literature and several techniques have been suggested for this purpose. The Canny, Sobel, Prewitt and Robert methods are some of the better-known edge detection techniques [12]. Each technique used for edge detection has advantages and disadvantages compared to others when analyzed in terms of the quality indicators of edge detection. In any case, perfect edge detection on real images is quite difficult because brightness and sharpness in gray level intensity that distinguishes objects from background in complicated images are not apparent. Since there are no certain conclusions within the studies introduced in the literature about perfect edge detection [13], there have been ongoing, extensive studies regarding the edge detection methods that produce almost perfect results [14,15]. Indeed, the operation principle of CNN is different from the operation of standard image processing techniques when it is interpreted in term of image processing. Due to the fact the mask parameter of well-known classical edge techniques is not used in the CNN structure, the CNN cloning template is designed according to the CNN structure. Design of the cloning template which determines the dynamic behavior of CNN is an important difficulty because a generalized template design method does not exist. Various methods have been proposed to determine such templates. These methods can be classified as analytical methods [16–19], local learning algorithms [20,21] and global learning algorithms [22,23]. The difficulty level of the problem increases depending on the number of variables and the type of data in all developed methods. However the solution for such problems using deterministic methods includes difficulties in both the modeling and solution processes, depending on the structure of the problem. Heuristic methods have been developed in order to overcome these disadvantages and produce a general solution that does not depend on the problem structure. The heuristic methods, which are based on population, can reach a solution fast, due to multiple search procedures [24]. If an appropriate quality metric for use in the heuristic methods can be defined, the optimal cloning template of CNNs, that must be adjusted correctly to realize the desired image processing practice, can be designed effectively by using the heuristic algorithms. Determining the cloning template of CNNs has been dealt with as an important optimization study and therefore several artificial intelligence based methods (such as Particle Swarm Optimization (PSO), Genetic Algorithm (GA), Differential Evolution (DE), *etc*.) have been proposed [25–33]. However, it is a shortcoming of these studies that results have not been assessed or compared visually or numerically with known classical techniques and previous studies made in the CNN area. It is required that the problem to be solved and the artificial intelligent optimization method should be in conformity and the control parameters of the optimization method should be set effectively. Since the results obtained with a single run are not sufficient to give a reliable interpretation of the technique used and the results obtained, determining optimal control parameter values of the optimization algorithm is possible by examining of results obtained with multiple running on different control parameter values of optimization. In this way, an idea can be suggested about whether the optimization technique selected is appropriate for the problem.

The Artificial Bee Colony (ABC) algorithm, inspired by the foraging behavior of honeybees, is a recently developed optimization algorithm [34]. For several problems, quite successful results have been obtained by using the ABC algorithm [35]. In addition to the successful results, it has been observed that the application areas of ABC increase rapidly due to its aspects such as easy applicability, less dependence on problem type, sensitive research in neighboring space, balancing of exploration and exploitation properties adaptively, producing effective and robust results regardless from the initial conditions at any time and fast convergence to the optimal solution when using only a few control parameters [36–38].

Due to the lack of a robust and effective structure in determining the cloning template, template designing studies are one of the most attractive research areas in the CNN field and this study aims to design the edge detecting CNN template through ABC algorithm. To examine the effects of ABC control parameters, performance analysis is made with multiple runs and the most effective control parameters for this application are identified by means of the data produced. The design template from the obtained results of ABC in this study is compared with other heuristic method-based CNN templates. The results are visually and quantitatively compared with the well-known classical edge detection methods and the other CNN based edge detectors in the literature by using artificial and real images.

The organization of the paper is as follows: firstly the CNN structure, cell concept and cloning template are explained in Section 2. Secondly, in Section 3, the structure of ABC is briefly described. Section 4 includes the information about how the training image used in the optimization is acquired. Optimization mechanism used to design cloning template of CNN by means of ABC for this application is presented in this section. Edge detection is made on different images and the results are compared with those of the widely used edge detection methods and the other CNN based cloning template in literature. The advantages of the results obtained by means of ABC are determined using several comparison metrics. Lastly, conclusions and suggestions are provided in Section 5.

## 2. Cellular Neural Networks (CNNs)

**Figure 1.**
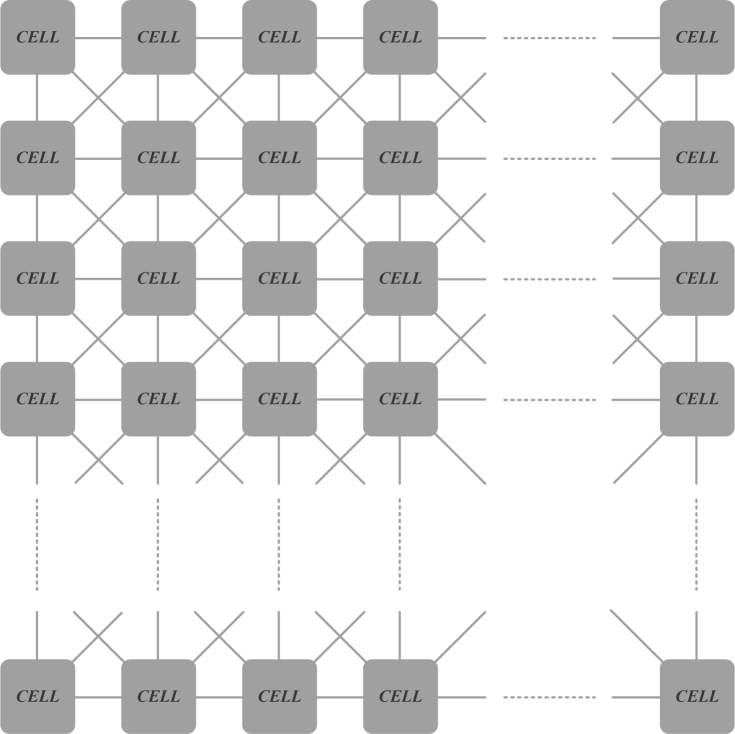
A general architectural structure of a CNN.

In this section, the structure of CNNs is briefly described. The two-dimensional (2D) network of a CNN structure is formed by the connection of “*cells*”. Each *cell* is connected only to its neighbor cells, in contrast to artificial neural networks [1–3]. This 2D network structure is also convenient for parallel processing applications and real time signal processing. With its capability of parallel processing, a CNN can easily perform image processing applications which pose a heavy load in terms of time and operation. Differently from a general Central Processor Unit (CPU), the CNN runs in parallel on its cells. CNN has many advantages over others in term of speed and capability. A general architectural structure for CNNs is shown in Figure 1.

As shown in Figure 1, each cell in a CNN is directly connected only with the neighbor cells. Due to the regional inner-cell connections in CNNs, a cell directly affects only its neighbor cells. Cells which are not directly connected to this cell are indirectly affected while transiting from the initial phase to a stable phase as a result of the propagation of CNN’s continuous time dynamics [1]. The cell concept and the cloning template terms are defined in the following subsections.

### 2.1. Cell

**Figure 2.**
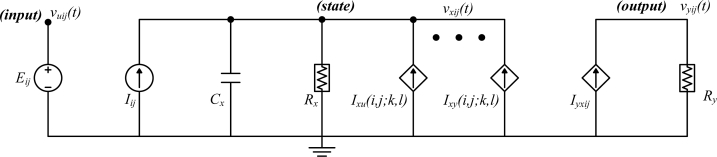
The circuit of a cell.

The cell, which is the basic element of the CNN structure, is composed of structurally linear and non-linear circuit elements, such as capacitors, linear resistances, linear and non-linear controlled sources and independent sources. The first CNN cell structure in the literature proposed by Chua [1] is shown in Figure 2.

In Figure 2, *E**_ij_* is the independent voltage source, *I_ij_* is the independent current source, *C_x_* is the capacitor, *R_x_* and *R_y_* are linear resistors, *I_xu_*(*i*,*j;k*,*l*) and *I_xy_*(*i*,*j;k*,*l*) are linear voltage controlled current sources with the characteristics of *I_xu_*(*i*,*j;k*,*l*) = *B*(*i*,*j;k*,*l*)*v_ukl_* and *I_xy_*(*i*,*j;k*,*l*) = *A*(*i*,*j;k*,*l*)*v_ykl_*, respectively. *i* and *j* indicate inner-cell index and *k* and *l* are neighborhood indexes of *M* × *N* size network. *v_xij_* and *I_ij_* shows the state voltage and bias current values of inner-cell (*C*(*i*,*j*)), respectively. These templates are described more clearly in Section 2.2. The only nonlinear element is piecewise linear voltage controlled current source with the characteristics of $I_{yxij}=1/2R_{y}(|v_{xij}+1|-|v_{xij}-1|)$.

In Equation (1), the radius (*r*) neighborhood of a *C*(*i*,*j*) cell in the CNN is given:

(1) $$N_{r}(i,\;j)=\{C(k,l)|\text{max}\{|k-i|,|l-j|\}\leq r,\;\;\;\;1\leq k\leq M;1\leq l\leq N\}$$

When the symmetry property is applied to all CNN, if *C*(*i*,*j*) ∈ *N_r_*(*k*,*l*), then *C*(*k*,*l*) ∈ *N_r_*(*i*,*j*), for all *C(i*,*j)* and *C(k*,*l)* cells. For a CNN, the time dependent (*t*) situations and output formulas are given in Equations (2) and (3), respectively:

*State Equation:*

(2) $$C\frac{dv_{xij}(t)}{dt}=-\frac{1}{R_{x}}v_{xij}(t)+\sum_{C(k,l)\in N_{r}(i,j)}A(i,j;k,l)v_{ykl}(t)+\sum_{C(k,l)\in N_{r}(i,j)}B(i,j;k,l)v_{ukl}(t)+I_{ij}\;\;\;\;\;\;\;\;1\leq i\leq M;1\leq j\leq N$$

*Output Equation:*

(3) $$v_{yij}(t)=\frac{1}{2}\left(\left|v_{xij}(t)+1\right|-\left|v_{xij}(t)-1\right|\right),\;\;\;\;\;\;\;\;\;\;1\leq i\leq M;1\leq j\leq N$$

In Equation (2), *v_ukl_*(*t*) and *v_ykl_*(*t*) are the input voltage and output voltage of the (*k,l*)th neighbor cell. *v_xij_*(*t*) and *I_ij_* as described above are the state and the bias current values of the cell *C*(*i*,*j*), respectively. *v_yij_*(*t*) given in Equation (3) is the output voltage equation of piecewise linear function and it is called the output function. In image processing applications, the input and output voltage of any cell represent the values of same index number pixel of input image and output image, respectively. The operation limit of a CNN is between –*1* and *1*, while any pixel value of an image changes from *1* to *255*. Therefore, pixel values of image are converted into operation limits of the CNN. Eventually, (–*1*) and (*1*) values indicate ‘*black pixel*’ and ‘*white pixel*’, and middle values represent ‘*gray-level pixel*’. In other words, *v_uij_*(*t*) and *v_ukl_*(*t*) indicates values of inner pixel and neighbor pixel in input image while *v_yij_*(*t*) and *v_ykl_*(*t*) indicates value of inner pixel and neighbor pixel in output image, respectively.

### 2.2. CNN Cloning Template

The dynamic behavior of a CNN is determined by the cloning template that can be considered as the indirect ratio of the inter-cell connections. The feedback cloning template *A*(*i*,*j;k*,*l*), the feed-forward cloning template *B*(*i*,*j;k*,*l*) and threshold cloning template *I*(*i*,*j*) given in Equation (2), form the cloning template for a cell. The feedback and feed-forward template are related with the outputs and inputs of neighbor cells. However, the threshold template is only related with the inner-cell. The *C*(*i*,*j*) cell is connected to neighboring cells at a ratio determined by these cloning template. Realizing the desired image processing can be done by arranging the values of this cloning template appropriately. For the *r = 1* neighborhood of the cell, the matrix structure of the cloning template *A*(*i*,*j;k*,*l*), *B*(*i*,*j;k*,*l*) and *I*(*i*,*j*) are given in Equation (4).

(4) $$A(i,j;k,l)=\left[\begin{array}{lll} a_{i-1,j-1} & a_{i-1,j} & a_{i-1,j+1}\\ a_{i,j-1} & a_{i,j} & a_{i,j+1}\\ a_{i+1,j-1} & a_{i+1,j} & a_{i+1,j+1} \end{array}\right]\;\;\;\;B(i,j;k,l)=\left[\begin{array}{lll} b_{i-1,j-1} & b_{i-1,j} & b_{i-1,j+1}\\ b_{i,j-1} & b_{i,j} & b_{i,j+1}\\ b_{i+1,j-1} & b_{i+1,j} & b_{i+1,j+1} \end{array}\right]\;\;\;\;\;\;I(i,j)=z(i,j)$$

The statement of Equation (4) can be stated as a general definition as in Equation (5):

(5) $$A=\left[\begin{array}{lll} a_{1} & a_{2} & a_{3}\\ a_{4} & a_{5} & a_{6}\\ a_{7} & a_{8} & a_{9} \end{array}\right]\;\;\;\;\;\;\;\;\;\;\;\;\;\;B=\left[\begin{array}{lll} b_{1} & b_{2} & b_{3}\\ b_{4} & b_{5} & b_{6}\\ b_{7} & b_{8} & b_{9} \end{array}\right]\;\;\;\;\;\;\;\;I=z$$

where *A*, *B* and *I* are represented as the template set, and this set, which is designed for the desired application, is given in Equation (6) as a vector:

(6) $$x_{i}=[a_{1},\;a_{2},\;a_{3},\;a_{4},\;a_{5},\;a_{6},\;a_{7},\;a_{8},\;a_{9},\;b_{1},\;b_{2},\;b_{3},\;b_{4},\;b_{5},\;b_{6},\;b_{7},\;b_{8},\;b_{9},\;z]$$

If the network cloning template of CNN can be arranged according to its purpose, the CNN can realize the desired simultaneous image processing successfully due to its ability to perform parallel processing. In the following section, the desired *x_i_* template set is obtained by using the ABC optimization algorithm for realizing edge detection processes on a CNN structure. The optimization structure and architecture of ABC algorithm are defined the following section.

## 3. Artificial Bee Colony (ABC) Algorithm

The Artificial Bee Colony (ABC) algorithm is inspired by the foraging behavior of honeybees. The most prominent properties of an ABC algorithm are easy applicability, less dependence on problem type, sensitive research, balancing of exploration and exploitation properties adaptively, producing effective and robust results with using only a few control parameters. In this section, the working mechanism of the ABC is presented and explained as a new approach to design cloning templates in this study.

### 3.1. Basic Principles of ABC Algorithm

This algorithm is developed based on the foraging principle of honeybees. There are three types of foraging bees in a honey bee colony; employed bees, onlooker bees and the scout bees. At the first stage, half of the colony is composed as employed bees (*n_e_*) and the other half is composed as onlooker bees (*n_o_*). There is only one employed bee for each nectar source. Nectar sources symbolize possible solutions. That is, the number of employed bees is equal to the number of nectar sources. The basic steps of the algorithm can be stated as follows:

**Step 1:** *Assign the control parameter values*
**Step 2:** *Initialize the population of solutions*
**Step 3:** ***Repeat as long as the stopping criteria is met***
  - Send the employed bees to the sources (solutions) and calculate their nectar amounts
  - Send the onlooker bees to the sources (solutions) and calculate their nectar amounts
  - Send the scout bees to find new sources randomly
  - Memorize the best sources achieved so far
**Step 4:** ***Stop***

Each cycle is mainly composed of three phases. For the first phase, the employed bees are sent to the sources and the nectar amounts of the sources visited are calculated. For the second phase, onlooker bees are sent to their sources and their nectar amounts are determined. For the third phase, it is ensured that the scout bee is located on a randomly selected new source. A food source corresponds to a possible solution to the problem where optimization is being attempted. The nectar amount of any source represents the quality level of the solution represented by that source. There are scout bees searching randomly in each colony. These bees do not use any kind of preliminary knowledge while searching for food and the search procedure is completely random. In the ABC algorithm, one of the employed bees is selected and made scout bee. This selection process is made based on the “*limit*” parameter. If a solution representing a source couldn’t be developed with a certain number of trials, this source is abandoned and the employed bee visiting this source becomes the scout bee. The number of trials determined for abandoning the source is identified by the “*limit*” parameter. In a robust search process, both exploration and exploitation occur simultaneously. The employed and onlooker bees are in charge of exploiting sources while scout bees are responsible for the exploration process. The bees work to maximize the energy function, stating the amount of food brought to the hive at any given unit time. The selection possibility is based on a probability function. After watching the dance of the employed bees and selecting the source at its position with the calculated probability value, the onlooker bee determines a source within the neighborhood of this source and starts collecting the nectar of this source. The position of the selected neighbor is calculated. If the nectar amount of the source at the new position is more than old one, then the bee visits the hive and shares her information with others and new position is memorized. Otherwise, old position is still kept in memory. If the nectar source at the a position remained constant without improving through cycles defined by the number of the “*limit*” parameter, the source at this position is abandoned and the employed bee of that source becomes the scout bee, doing random research. The newly found source is assigned instead of abandoned position [36–38]. The mathematical description of the ABC algorithm is explained exhaustively in the following section.

### 3.2. Mathematical Description of ABC Algorithm

In this section, the working mechanism of ABC is presented mathematically. The position of a food source (*x_i_*) represents a feasible solution of problem and the amount of nectar of the food source indicates the fitness value of the associated solution in the ABC algorithm. The colony size of employed bees (*n_e_*) is equal to the colony size of onlooker bees (*n_o_*) in the population. A set of food sources positions (*x_1_,…, x_ne_*) is produced randomly:

(7) $$x_{i}=x_{j}^{\text{min}}+rand(0,1)\left(x_{j}^{\text{max}}-x_{j}^{\text{min}}\right)$$

where *i* = *1*,…,*SN* and *j* = *1*,..,*D. SN* is the number of food sources and *D* is number of optimization parameters. Also, it must be noted that *SN* = *n_e_* = *n_o_*. All counters associated with solutions are reset to 0 in this phase. The colony of employed bees can be expressed by *n_e_* dimension vector *x⃗*(*n*) = (*x*_1_ (*n*), ..., *x*_*n_e_*_ (*n*)), where *n* is cycle value of ABC algorithm. In additional, individual search space can be signified as *S. x_i_* ∈ *S* and *i* ≤ *n_e_* in *x⃗*. After initialization, the fitness value of each solution is calculated and *x⃗*(0) is obtained. To evolve quality of solutions, employed bees change their position from the current position to neighboring source positions by using the following equation:

(8) $$v_{ij}=x_{ij}+\phi_{ij}(x_{ij}-x_{kj})$$

where *j* ∈ {1, ..., *D*}, *k* ∈ {1, ..., *SN*}, *k* ≠ *j*, *j* and *k* are randomly chosen index. Ø*_ij_* is a real number produced randomly in the range [–1,1]. Values of parameters produced in this process are within in determined boundaries (*v* ∈ *S* and *S* → *S*). After producing *v_i_*, to select better solution for next generation, greedy selection operator is applied. Probability distribution of this operator can be given as follows:

(9) $$P\{x_{i},\;v_{i}\}=\{\begin{array}{ll} 1, & f(v_{i})\geq f(x_{i})\\ 0, & f(v_{i})<f(x_{i}) \end{array}$$

where *f*(*v_i_*) and *f*(*x_i_*) are nectar amounts of food sources at *v_i_* and *x_i_*, respectively. If *v_i_* is better solution than *x_i_*, the employed bee memorizes position of *v_i_*, otherwise position of *x_i_* is retained. In the end of this process, if a better solution cannot be obtained, the trials counter associated with the solution is incremented by 1, and otherwise it is reset to 0.

After the employed bees phase is completed, the phase of onlooker bees is started by selecting an employed bee from the colony. The probability of the selection process depends on the fitness values of the solutions and many selection schemas such as roulette wheel and stochastic universal sampling can be used. Probabilistic function of roulette wheel mostly used ABC can be described as follows:

(10) $$P\{x_{i}\}=\frac{f(x_{i})}{\sum_{i=1}^{SN}f(x_{i})}$$

This function means that, if the fitness value of a solution increases, the visiting number of onlooker bees to that source increases too. To decide if a modification will be made on an onlooker bee position, a random real number within the range [0,1] is generated for each source. If the generated number is less than the probability value in Equation (10), then the onlooker bee changes position by using Equation (8) to find new solutions. Greedy selection is applied to the modified source and then if the new position is better than the old position, the memory of the onlooker bee is updated, otherwise the old position is kept. According to the result of this process, the counter associated with onlooker bees is incremented by 1 or reset to 0 similar to the operation in the employed bee phase.

To end a cycle, if a counter value of employed bees and onlooker bees reaches its “*limit*” value, the source of this counter is abandoned. A new food source is discovered by the scout bee and it replaces the abandoned source. This operation can be defined as follows:

(11) $$x_{i}(n+1)=\{\begin{array}{ll} x_{\text{min}}+\text{rand}(0,1)(x_{\text{max}}-x_{\text{min}}), & \text{counter}\geq \text{limit}\\ x_{i}(n), & \text{counter}<\text{limit} \end{array}$$

This operation is a noticeable property of ABC algorithms because this operation is different from other algorithms and it can improve the search efficiency of the ABC. A detail theoretical explanation about convergence and complexity analysis of ABC can be found in [39]. ABC optimization study is explained exhaustively in order to realize proposed application by using ABC in the following section.

## 4. Proposed Method

It is the aim of this study to effectively determine the edge detection cloning template of the CNN with an optimization mechanism set up using suitable quality metric and training images. In this section, the training image used in optimization, the quality metrics and the optimization study are explained.

### 4.1. Preparation of Training Images for ABC Optimization

Ideal edge detection of a noise free image which has homogenous objects or regions is achieved by using the pseudo code given below. The image obtained by using this method is used as the desired output image of the proposed method in this paper:

start

for i=1:m

  *for j=1:n*

    *if eP_i,j_* *< eP_i+1,j_* *then dP_i,j_=1*

    *if eP_i,j_* *< eP_i,j+1_* *then dP_i,j_=1*

    *if eP_i,j_* *> eP_i+1,j_* *then dP_i+1,j_=1*

    *if eP_i,j_* *> eP_i,j+1_* *then dP_i,j+1_=1*

  *end*

end

**Figure 3.**
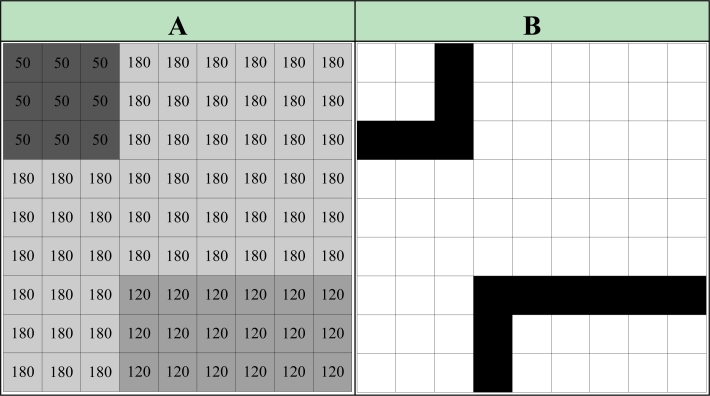
**(a)** A part of the input image **(b)** The ideal edge detected image.

Here, *i* and *j* indicate the coordinate index of the pixels in the image, *eP* indicates the pixel value of the training input image, *dP* indicates the pixel value of the training output image. In order to explain how the Pseudo code works, a part of the pixel values of an artificial image given in Figure 3(a) is used. The lines with pixel changes is detected using the given pseudo code and the ideal edge detection in Figure 3(b) is made and the result is obtained. It is important that the lines be determined in one pixel thinness and with continuous lines for the edge detection quality.

**Figure 4.**
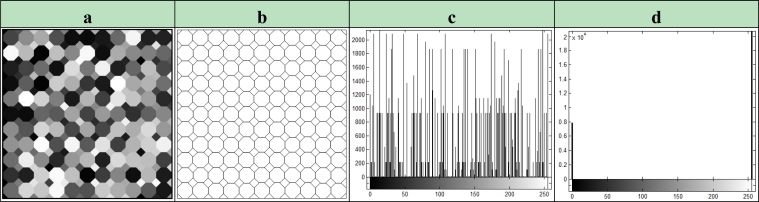
**(a)** Training input image. **(b)** The ideal edge detected image of training image. **(c)** Histogram of training input image. **(d)** Histogram of edge detected image.

The image of *374* × *374* pixel size given in Figure 4(a) is used as the training input image. The training input image is an artificial image that is formed in a way that it covered 62% of the grayscale. Each octahedral part has a homogeneous area with the same pixel value. As explained in above, the ideal edge detection process is performed by determining the edge lines where homogeneous octahedral areas intersect. Hence, the desired output image is obtained, which is given in Figure 4(b). Moreover, the image histograms of input and desired images are demonstrated in Figure 4(c,d), respectively.

### 4.2. Designing the CNN Cloning Template Using ABC Algorithm

In this section, the assumptions and the optimization structure that are used to design the CNN’s cloning template are explained. In order to guarantee the stability of the CNN, the symmetry assumption given in Equation (12) is applied to the cloning template defined in Equation (5) and eventually Equations (13,14) are obtained [3]. As a result, the duration of the optimization is reduced by decreasing the template parameters to be optimized and additionally it is ensured that stable outputs can be acquired for the CNN:

(12) $$\begin{array}{l} A(i,j;k,l)=A(k,l;i,j)\\ B(i,j;k,l)=B(k,l;i,j) \end{array}\}|x_{ij}(0)|\leq 1,\;\;\;\;|u_{ij}|\leq 1,\;\;\;\;\;\;1\leq i\leq M;1\leq j\leq N$$

(13) $$A=\left[\begin{array}{lll} a_{1} & a_{2} & a_{3}\\ a_{4} & a_{5} & a_{4}\\ a_{3} & a_{2} & a_{1} \end{array}\right]\;\;\;\;\;B=\left[\begin{array}{lll} b_{1} & b_{2} & b_{3}\\ b_{4} & b_{5} & b_{4}\\ b_{3} & b_{2} & b_{1} \end{array}\right]\;\;\;I=z$$

(14) $$x_{i}=[a_{1},\;a_{2},\;a_{3},\;a_{4},\;a_{5},\;b_{1},\;b_{2},\;b_{3,}\;b_{4},\;b_{5},\;z]$$

**Figure 5.**
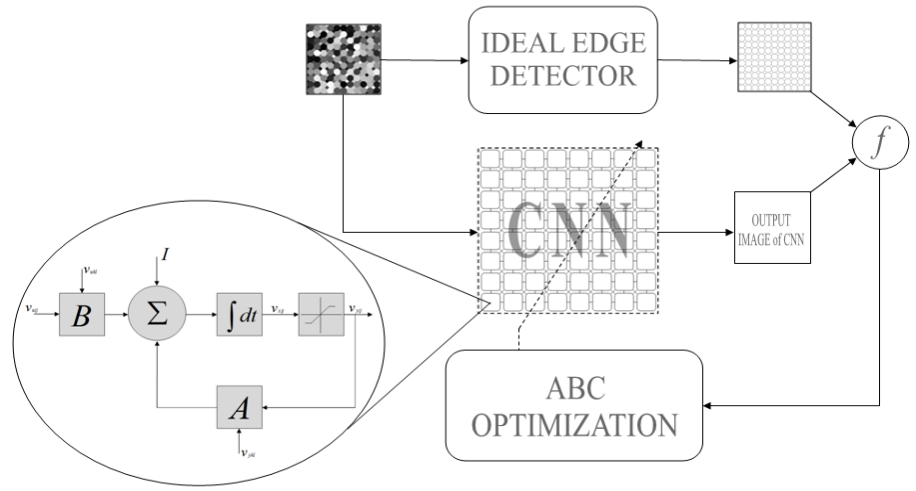
Template design mechanism of the ABC based CNN edge detector.

The ABC design mechanism of the cloning template is shown in Figure 5. In this mechanism, the output image of the CNN converges to the desired image by adjusting the cloning template of the CNN by means of the ABC algorithm. The ABC algorithm produces template set and sends this set to the the CNN. CNN runs by using this set and training image, and it generates an output image. The fitness value of the objective function is calculated by comparing the output image of the CNN and the ideal edge detected image. The ABC algorithm evaluates with this fitness value in order to obtain better results.

Running of ABC optimization can be explained as follows: a *D* × *SN* size matrix is randomly selected as the initial colony and each row of the matrix represents a cloning template set, a possible solution to the problem, in the ABC optimization. *D* is the dimension of the problem and *SN* is the size of the colony. There are three main phases in the ABC algorithm; the employed bees phase, the onlooker bees phase and the scout bees phase.

The fitness value of each bee in the employed bee’s colony (*n_e_*) is calculated by using Equation (16) which is based on the correlation between output image of the CNN mechanism and the desired output Figure 4(b). Local search is applied by Equation (10) at neighbors of all existing cloning templates. If better fitness values are obtained after the local search process, these templates are included in the population and the employed bees phase is completed. The onlooker bees phase is similar to the previous phase. The only difference from the other is the operation of the local search mechanism. This operation is not applied to all template sets, it is applied to some template set which is the probability selected with the roulette wheel. Thus, the selection of the worse template is as much possible as selection of a better template and therefore diversity in the population is ensured. If a solution is not improved in both phases, the testing counter that is related with the solution is incremented by 1, otherwise the counter reset to 0. In the scout bees phase, which template is removed from population is arranged by controlling the testing counters by comparing these counters with the parameter named “*limit*”. If the testing counter value of a template set reaches the *limit* value, this set is removed from the population and a new cloning template set produced randomly is replaced instead of the removed templates. All the explained processes are repeated as many times as indicated by the as number of cycles of the ABC:

(15) $$C=\frac{\sum_{i}^{m}\sum_{j}^{n}\left(zP_{ij}-\bar{zP})(tP_{ij}-\bar{tP}\right)}{\sqrt{\left(\sum_{i}^{m}\sum_{j}^{n}{\left(zP_{ij}-\bar{zP}\right)}^{2}\right)\left(\sum_{i}^{m}\sum_{j}^{n}{\left(tP_{ij}-\bar{tP}\right)}^{2}\right)}}$$

(16) $$f(x_{i})=\frac{1}{C+ɛ},\;\;\;\;\;\;\;\;\;0\leq r\leq 1$$

where *i* and *j* are pixel index on the image, *zP* is pixel value of the CNN output image, *tP* is pixel value of the edge detected training image, $\bar{zP}$ is mean of the output image, $\bar{tP}$ is mean of the edge detected training image, ɛ is a small positive constant, and *f* is objective function. The *C* value in Equation (16) is the correlation between the two images and this is given in Equation (15).

By calculating the similarity between the output images of the CNN and the ideal edge detected image, the optimization process is continued to prove better results by the ABC algorithm. Simulations are developed on a MATLAB platform and the processing of the CNN structure is realized using the MATCNN library [40,41].

### 4.3. The Simulation Results

The performance and robustness of ABC algorithm in determining the edge detecting CNN cloning template are assessed statistically. The optimization results are obtained with multiple runs on different control parameters of ABC; “*limit*” and the “*size of colony*” (*NP*), which are the control parameters of ABC, are considered in the simulations [36].

In order to show the effect of the scout production mechanism on the performance of the algorithm, the average of the best function values found for the different “*limit*” values (*1* × *n_e_* × *D*, *2* × *n_e_* × *D*, *4* × *n_e_* × *D* and “*without scout*”) and colony sizes (*20*, *40* and *80*) is given in Table 1. In the simulations, the cycle and time step constant of the CNN are selected as *30* and *0.3*, respectively. Three thousand cycles are set for each combination of *NP*, and “*limit*” value and ABC is run independently *30* times and the results obtained (*f* values in Equation (16)) are given in Table 1 as mean (*MEAN*) and standard deviation (*STD*).

In Table 1, it is seen that *STD* values are very low in all combinations of *NP* and “*limit*” values. Therefore, it can be stated that the ABC optimization technique for this problem is quite a robust algorithm. Moreover, the fact that the *MEAN* value gets smaller as the *n* value gets bigger supports the assumption that choosing a smaller *n* value can lead to better results. Beside the results of Table 1, in order to present a stable evaluation of the effect of control parameters of the ABC on its performance, ANalysis Of the VAriance (ANOVA) based on the mean variance is used. ANOVA examines the statistical difference of one, two or more groups over one quantitative one. The null hypothesis we established for ANOVA is that all population means are equal and the other hypothesis is that at least one mean is different from the others. Calculated F value is compared to the value coming from the F distribution associated with the degrees of freedom and significance value. If this value is lower than 0.05, the effect of the variable is considered to be statistically significant. ANOVA statistics are calculated by using 12 different values of control parameters on MATLAB platform. Each data group has 30 *f* values coming from each run. Results of ANOVA test for *NP* and *limit* control parameters are presented in Table 2. In the table, each column presents values of the sum of squares (SS), the degrees of freedom (df), Mean Squares (MS), which is the ratio SS/df and F statistics, which is the ratio of the mean squares. From the results in Table 2, it can be said that *f* function is influenced significantly by the values of *NP* and *limit*.

At the values of *MEAN = 0.7935* and *STD = 0.0521* where optimization is at its best and most effective, the population size and *limit* values are *80* and *440*, respectively. The best *C* value obtained so far is *C = 0.9331* and the cloning template obtained that correspond to this value are presented in Equation (17):

(17) $$A=\left[\begin{array}{lll} -\mathrm{0.2481} & -\mathrm{5.4392} & -\mathrm{1.2947}\\ -\mathrm{7.3106} & \mathrm{62.7200} & -\mathrm{7.3106}\\ -\mathrm{1.2947} & -\mathrm{5.4392} & -\mathrm{0.2481} \end{array}\right]\;\;\;\;B=\left[\begin{array}{ccc} -\mathrm{0.0051} & \mathrm{0.0610} & \mathrm{0.1331}\\ -\mathrm{0.0739} & -\mathrm{34.2720} & -\mathrm{0.0739}\\ \mathrm{0.1331} & \mathrm{0.0610} & -\mathrm{0.0051} \end{array}\right]\;\;\;\;I=-\mathrm{1.6937}$$

The cloning template given in Equation (17) is the optimal template designed by the ABC algorithm in this study for the purpose of edge detection.

### 4.4. Related Works

In this section, cloning templates of previous studies are presented and used to compare with the ABC results. Designing the cloning template of CNN is an important task to achieve the desired results and several technique-based analytic methods, local learning algorithms and heuristic methods are proposed. Some of heuristic methods also used are Differential Evolution (DE) [27], Evolution Strategies (ES) [28] techniques and Genetic Algorithm (GA) [22,32]. The first studies in the CNN area were concentrated around binary images and later, some studies including gray-level images are presented. Most template design studies do not include performance analyses of heuristic methods for edge detection application and they are only introduced for design studies. In addition, templates presented in previous studies are not compared subjectively and quantitatively with other templates determined in the same area. Furthermore, edge detection studies based on CNN introduced without applying threshold process with grayscale real images first are very limited. Therefore, there are doubts about the reliability of heuristic methods in this kind of applications. In order to alleviate these deficiencies, a performance analysis of the ABC is performed with multiple runs using its different control parameters. The cloning template obtained with the ABC algorithm is compared subjectively and quantitatively with other well-known techniques and previous studies on designing CNN cloning templates. In the following tables, cloning templates determined in previous studies for edge detection applications are presented. One of the presented cloning templates is from the MATCNN Template Library (EDT-ML) [41]. The other cloning template is obtained by using the Differential Evolution method (DE-CNN) [27]. Another cloning template is introduced by using Evolution Strategies (ES-CNN) [28] and Xavier [29]. In the following sections, comparisons are made using these templates.

### 4.5. The Method of Comparison and Parameters

The edge detection quality of the templates in Equation (17), determined as a result of optimization, is assessed by using both artificial binary and grayscale images, and real images. Edge detection is performed on all images by using the well-known classical edge detector and the previously proposed CNN based edge detectors and the novel CNN templates in Equation (17).

**Figure 6.**
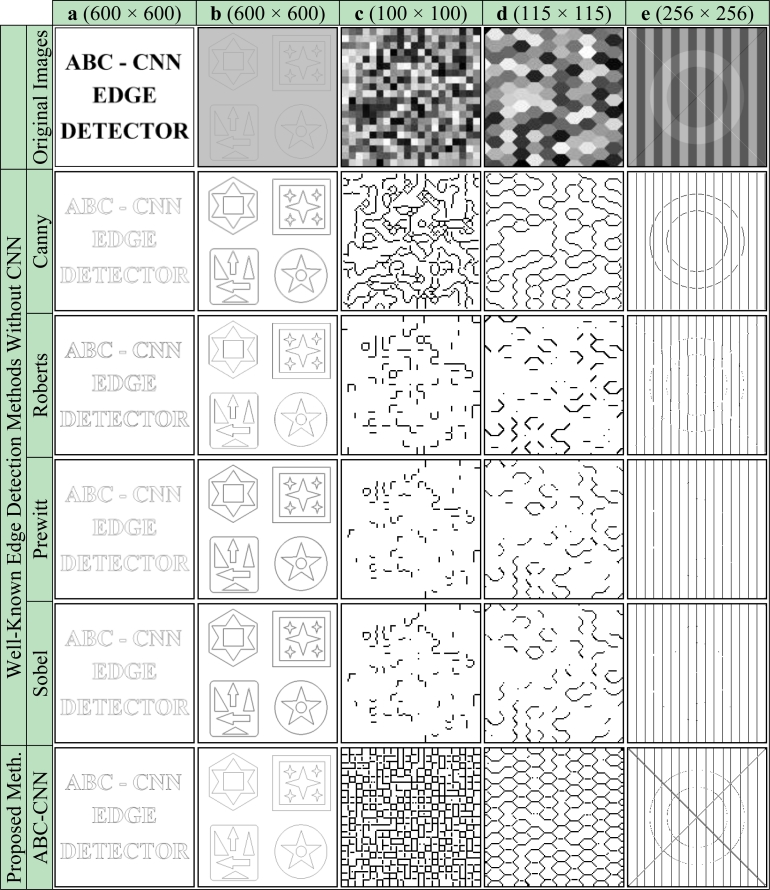
Artificial images. **(a)** Text. **(b)** Shape. **(c)** Check. **(d)** Honeycomb. **(e)** Ledge [42].

**Figure 7.**
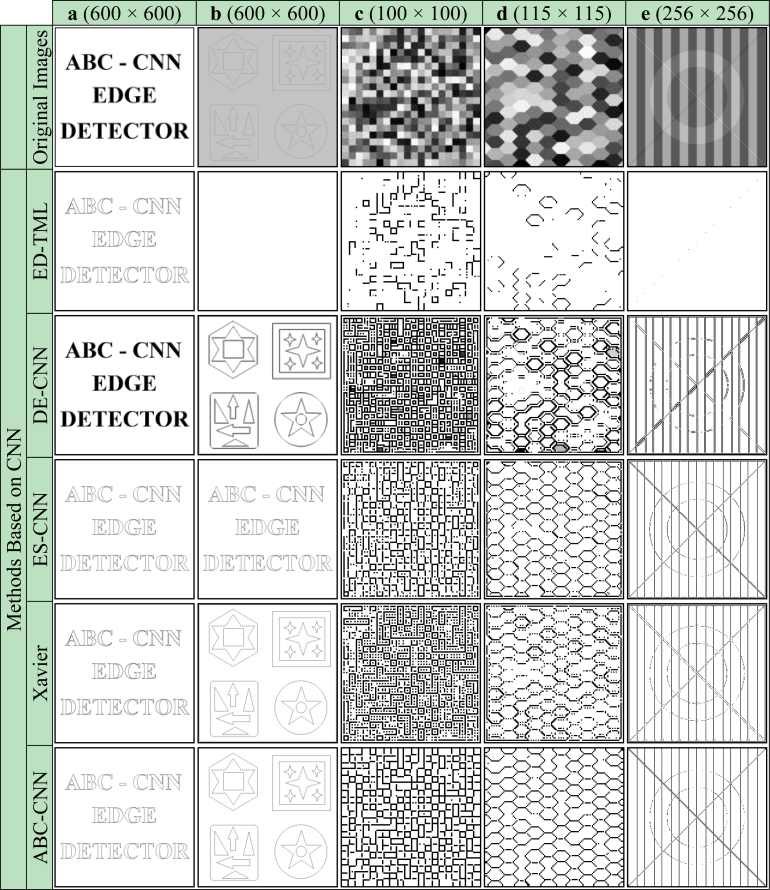
Artificial images. **(a)** Text. **(b)** Shape. **(c)** Check. **(d)** Honeycomb. **(e)** Ledge [42].

**Figure 8.**
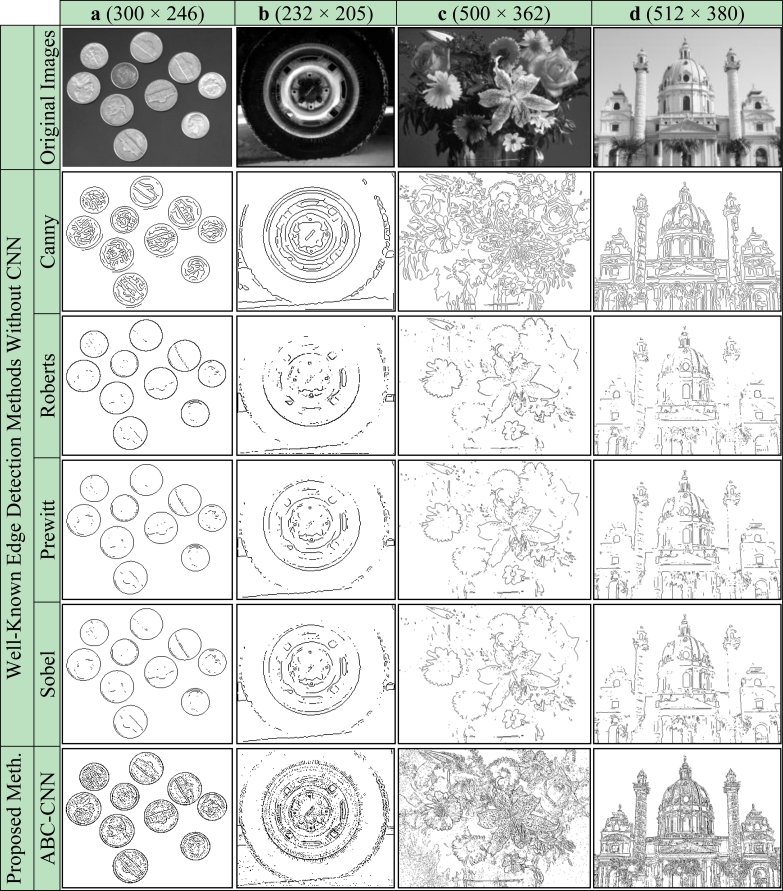
Real images. **(a)** Coin. **(b)** Wheel. **(c)** Flowers. **(d)** Church.

**Figure 9.**
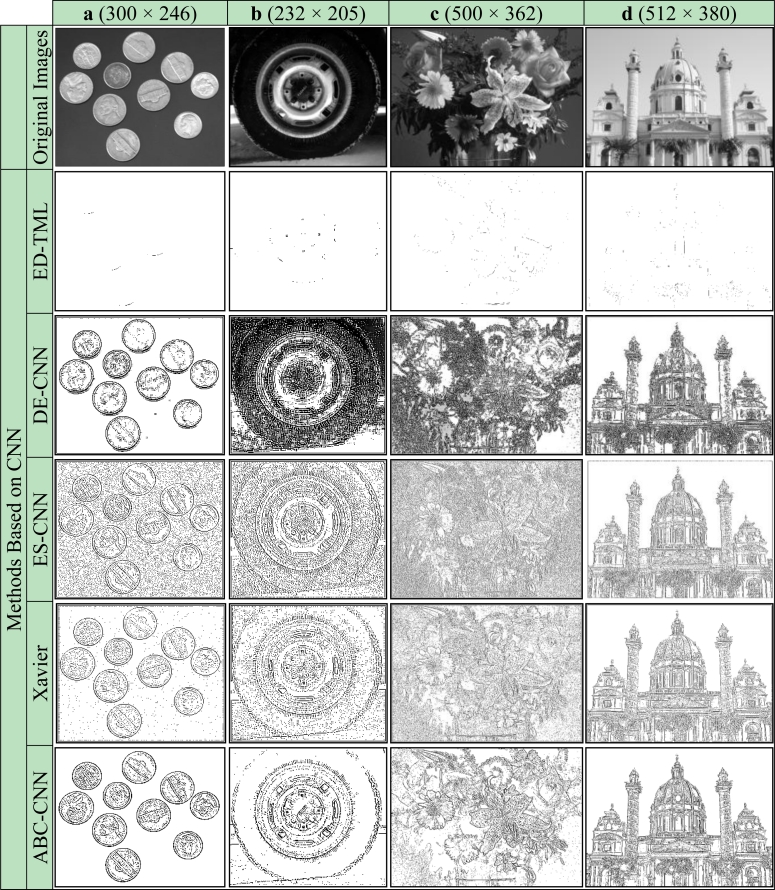
Real images. **(a)** Coin. **(b)** Wheel. **(c)** Flowers. **(d)** Church.

Firstly, subjective comparisons are presented in Figures 6–9. For better evaluation of these subjective comparisons, the proposed method is given at the end of the rows of figures. The study is continued with objective evaluation based on results of accepted comparison metrics.

Subjective comparisons of obtained cloning template and well-known edge detection methods by use of the binary/grayscale artificial images are given in Figure 6.

Figure 6(a) shows that all classic edge methods determined the ideal edges of the Text image successfully. For the Shape image, as can be seen in Figure 6(b), it is seen that compared with the proposed method the other methods are unsuccessful as indicated by the duplicate lines that occur in the output edge image. It is obvious that for the gray-level artificial images, the Check, Honeycomb and Ledge, as shown in Figure 6(c–e), respectively, the proposed method detected the edges as well as the ideal edge detection, and, in fact is considerably more successful than well-known edge detection methods. Subjective comparisons of obtained cloning template and previous studies based on CNN by use of the binary/grayscale artificial images are given in Figure 7.

Figure 7(a) shows that methods based on CNN, except for DE-CNN, determined the ideal edges of the Text image successfully. For the Shape image, as can be seen in Figure 7(b), ES-CNN templates, Xavier templates and ABC-CNN templates detects all boundaries as single lines. However the DT-CNN method is unsuccessful as indicated by the duplicate lines that occur in the output edge image and the results of the ED-TML method are unclear. It is obvious that for the gray-level artificial images, the Check, Honeycomb and Ledge, as shown in Figure 7(c–e), respectively, the proposed method detected the edges as well as the ideal and noiseless edge detection, and, in fact is considerably more successful than other methods. Subjective comparisons of obtained cloning template and well-known edge detection methods by use of the binary/grayscale real images are given in Figure 8.

For real images, Coin, Wheel, Flowers and Church, the edge detection results are given in Figure 8(a–d), respectively. As can be seen from Figure 8, the results of Roberts, Prewitt and Sobel are unsatisfactory. The results obtained by the Canny methods are a bit better than the others; however, the proposed ABC-based CNN method has the best results, with more details and less noise in the edge images. For example, the results of the Canny method have duplicate edge lines for the outer borders of the coins in Figure 8(a) and loss of details for Figure 8(b–d). Subjective comparisons of obtained cloning template and previous studies based on CNN by use of the binary/grayscale real images are given in Figure 9.

As can be seen the results of ED-TML are unsatisfactory. In the Xavier and ES-CNN method results, the output images have too much noise, which reduces the perception of the edge detection output. There are many undetected objects in the DE-CNN results, especially for Figure 9(b,c). It is obvious that the results of the proposed ABC-based CNN method have more distinctive details than other methods and less noise in the edge images.

**Figure 10.**
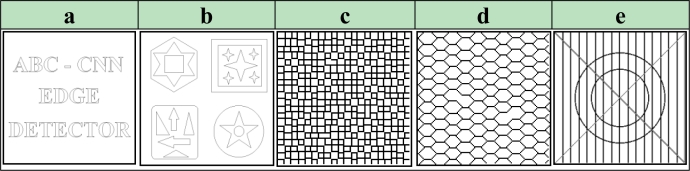
The results of the ideal edge detection obtained using the ideal edge method.

Besides visual evaluation of the edge detection quality on binary and grayscale artificial images, it is also the aim of the study to assess the edge detection quality quantitatively. For this reason, the similarity between the results shown in Figures 6 and 7 and the ideal edge detection results given in Figure 10 are compared.

Correlation (*C*), structural similarity (*SSIM*) [43] and mean squared error (*MSE*) values are used as the comparison operators and results are presented in Table 4. To calculate the correlation, the equation given in Equation (15) is used. Mean Squared Error (*MSE*) is given in Equation (18):

(18) $$MSE=\frac{1}{mn}\sum_{i=0}^{m-1}\sum_{j=0}^{n-1}{\left(zP(i,j)-tP(i,j)\right)}^{2}$$

where *m* × *n* is size of image, *i* and *j* are coordinate index of the pixels in the image. SSIM, which is another similarity operator used for comparison, is given in Equation (19) [43]:

(19) $$SSIM=\frac{\left(2\mu_{zR}\mu_{tR}+c_{1})\;(2\sigma_{zRtR}+c_{2}\right)}{\left(\mu_{zR}^{2}+\mu_{tR}^{2}+c_{1})\;(\sigma_{zR}^{2}+\sigma_{tR}^{2}+c_{2}\right)}$$

(20) $$c_{1}={\left(k_{1}L\right)}^{2},\;\;c_{2}={\left(k_{2}L\right)}^{2}$$

Here, *μ_zR_*, *μ_tR_* are the mean gray-level intensity values of *zR* and *tR* images, respectively. σ*_zR_* ^2^ and σ*_tR_* ^2^ are the variance of *zR* and *tR* images, respectively. *σ_zRtR_* is the covariance of *zR* and *tR* images. *L* is the dynamic interval of the pixel value and *k_1_* and *k_2_* are the constant numbers that are *0.01* and *0.03* [43].

The higher values for *C* and *SSIM*, and the lower values for *MSE* in Table 4 imply better results. As previously mentioned for the visual evaluation of the Text image results, all methods give better results. However, the quantitative results given in Table 4 show that some of the methods are unsuccessful in terms of *C* and *MSE*. This is because the output edge images have drifts along the edge borders in comparison to the ideal edge image. A similar situation can be seen on the results of other images. As a result, it is clearly seen from Table 4 that the ABC-based CNN method performs better than others in terms of *C*, *MSE* and *SSIM*. Also, these numerical results confirm the previously mentioned visual evaluations.

## 5. Conclusions

By using the ABC algorithm, optimal templates for edge detection were established. In this study, first, in order to inspect the suitability of ABC for this problem, a statistical evaluation of the algorithm was made. Based on the statistical results, it was seen that lower *STD* values show that ABC is a stable and robust algorithm, and higher *MEAN* values show that ABC optimization technique is a convenient technique for this problem. Moreover, the control parameter values where the algorithm gives the best results were detected as *NP = 80* and *limit = 440*. By using these control parameter values, the CNN cloning template used for edge detection were obtained. Next, the templates found were employed for edge detection of binary and grayscale artificial images and real images. Furthermore, edge detection results of well-known edge detection methods were obtained. As a result of the subjective evaluations, it was concluded that the results obtained through ABC-CNN templates were more appropriate. In order to confirm the subjective evaluations, quantitative evaluations were made with numerical comparisons by using the well-known *C*, *MSE* and *SSIM* functions in literature. Similar to the visual subjective evaluation, the quantitative evaluation results also suggest that the results obtained by ABC-CNN templates are more qualified.

The main contributions of this study can be summarized as follows:

- This is the first study employing ABC technique for determining CNN templates.
- In this study, the performance of ABC optimization in determining CNN templates was investigated statistically, due to the random nature of the heuristic algorithms.
- The results of the detected templates were assessed on binary and grayscale images. Visual and numerical comparisons are limited in the literature. In this study, both the results were compared visually and numerical comparisons were made in order to confirm the visual assessment.
- The results obtained with ABC-CNN templates gave quite successful outputs compared to the well-known classical edge detection techniques and the CNN templates reported in the literature.

## Figures and Tables

**Table 1.** The performance analysis results of ABC.

***Colony***	***NP = 20 (n****_e_****= n****_o_****= 10)***				***NP = 40 (n****_e_****= n****_o_****= 20)***				***NP = 80 (n****_e_****= n****_o_****= 40)***			
	
***Size***	***Limit = 1***×***n****_e_*×***D***	***Limit = 2***×***n****_e_*×***D***	***Limit = 4***×***n****_e_*×***D***	***Without scout***	***Limit = 1***×***n****_e_*×***D***	***Limit = 2***×***n****_e_*×***D***	***Limit = 4***×***n****_e_*×***D***	***Without Scout***	***Limit = 1***×***n****_e_*×***D***	***Limit = 2***×***n****_e_*×***D***	***Limit = 4***×***n****_e_*×***D***	***Without scout***
***MEAN***	*0.7551*	*0.7340*	*0.7013*	*0.4525*	*0.7625*	*0.7450*	*0.7172*	*0.6483*	***0.7935***	*0.7509*	*0.7656*	*0.7048*
***STD***	*0.0517*	*0.0642*	*0.1660*	*0.2904*	*0.0643*	*0.0860*	*0.1057*	*0.2031*	*0.0521*	*0.0456*	*0.0483*	*0.1192*

***n****_e_*: number of employed bees, *D*: dimension of problem, runs = 30 and total evaluation number = 3,000.

**Table 2.** ANOVA table for *f* function by 12 different control parameters of ABC.

**Function**	**SS**	**df**	**MS**	**F**	**Prob > F**
*f*	*2.65833*	*11*	*0.24167*	*14.21*	*1.22746e-022*

**Table 3.** Presented templates of previous studies based on CNN.

***METHODS***	***A TEMPLATE***	***B TEMPLATE***	***I TEMPLATE***	***PARAMETER OF CNN***
***ED-TML[41]***	$\left[\begin{array}{ccc} 0 & 0 & 0\\ 0 & 2 & 0\\ 0 & 0 & 0 \end{array}\right]$	$\left[\begin{array}{ccc} -0.25 & -0.25 & -0.25\\ -0.25 & 2 & -0.25\\ -0.25 & -0.25 & -0.25 \end{array}\right]$	−1.5	*Time Step = 0.3* *Iteration = 50*
***DE-CNN[27]***	$\left[\begin{array}{ccc} -5.9791 & -2.3636 & -5.9905\\ -0.5346 & -14.8176 & -0.5346\\ -5.9905 & -2.3636 & -5.9791 \end{array}\right]$	$\left[\begin{array}{ccc} 0.0304 & -0.0198 & 0.0049\\ -0.0453 & 15.0325 & -0.0453\\ 0.0049 & -0.0198 & 0.0304 \end{array}\right]$	1.1570	*Time Step = 1.3937* *Iteration = 2*
***ES-CNN[28]***	$\left[\begin{array}{ccc} -0.0188 & -7.2196 & -1.6024\\ -2.2306 & 20.8999 & -2.2306\\ -1.6024 & -7.2196 & -0.0188 \end{array}\right]$	$\left[\begin{array}{ccc} -0.0397 & 0.3402 & -0.0362\\ -0.2233 & -0.2497 & -0.2233\\ -0.0362 & 0.3402 & -0.0397 \end{array}\right]$	−3.3014	*Time Step = 0.3* *Iteration = 20*
***Xavier[29]***	$\left[\begin{array}{ccc} -0.2367 & -0.2416 & -0.2207\\ -0.1710 & 1.8889 & -0.1710\\ -0.2207 & -0.2416 & -0.2367 \end{array}\right]$	$\left[\begin{array}{ccc} -0.6522 & 0.0306 & -0.8290\\ -0.0722 & 2.8511 & -0.0722\\ -0.8290 & 0.0306 & -0.6522 \end{array}\right]$	−1.3631	*Not given in the paper, the values used: Time Step = 0.03* *Iteration = 100*

**Table 4.** Comparison results of the detection methods with the results of ideal edge detection for the binary and grayscale artificial images given in Figure 6.

	***TEXT***			***SHAPE***			***CHECK***			***HONEYCOMP***			***LEDGE***		

	***C***	***MSE***	***SSIM***	***C***	***MSE***	***SSIM***	***C***	***MSE***	***SSIM***	***C***	***MSE***	***SSIM***	***C***	***MSE***	***SSIM***
***CANNY***	*0.3393*	*1589.5*	*0.8733*	*0.0265*	*4254.3*	*0.6755*	*0.1922*	*20470*	*0.1774*	*0.3304*	*9228.9*	*0.3005*	*0.6393*	*5432.3*	*0.6335*
***ROBERTS***	*0.5062*	*1334.8*	*0.8951*	*0.6908*	*1048.3*	*0.9038*	*0.0867*	*21211*	*0.0532*	*0.2081*	*9568.1*	*0.1547*	*0.5281*	*6746.0*	*0.5432*
***PREWITT***	*0.4709*	*1260.0*	*0.8950*	*0.0260*	*4337.7*	*0.6806*	*0.0814*	*21055*	*0.0498*	*0.2001*	*9332.1*	*0.1484*	*0.5812*	*6163.6*	*0.5855*
***SOBEL***	*0.4602*	*1309.5*	*0.8929*	*0.0285*	*4300.5*	*0.6833*	*0.0805*	*21081*	*0.0493*	*0.2445*	*8973.2*	*0.1779*	*0.5819*	*6155.6*	*0.5859*
***ED-TML***	*0.9209*	*217.1*	*0.9855*	*0.0001*	*1456.4*	*0.7669*	*0.4428*	*15527*	*0.3411*	*0.4355*	*7154.3*	*0.2805*	*0.0324*	*9874.3*	*0.2507*
***DE-CNN***	*0.4545*	*4351.2*	*0.8914*	*0.5863*	*2607.0*	*0.7720*	*0.5736*	*14037*	*0.5946*	*0.5081*	*10093.*	*0.4975*	*0.3885*	*10060.*	*0.3500*
***ES-CNN***	*0.9632*	*94.6*	*0.9926*	*0.8735*	*436.0*	*0.9898*	*0.7461*	*7120*	*0.7693*	*0.7798*	*3456.5*	*0.8675*	*0.6382*	*5427.3*	*0.6819*
***XAVIER***	*0.9059*	*263.1*	*0.9825*	*0.8730*	*436.7*	*0.9899*	*0.7251*	*7547*	*0.7780*	*0.6224*	*5686.0*	*0.6947*	*0.5615*	*6356.8*	*0.6106*
***ABC-CNN***	***0.9754***	***62.0***	***0.9954***	***1.0000***	***0.0***	***1.0000***	***0.8609***	***4006***	***0.8614***	***0.8936***	***1622.6***	***0.9160***	***0.6610***	***5157.5***	***0.6768***
